# Establishment of Toxicity and Susceptibility Baseline of Broflanilide for *Aphis gossypii* Glove

**DOI:** 10.3390/insects13111033

**Published:** 2022-11-08

**Authors:** Ren Li, Shenhang Cheng, Zhibin Chen, Tianfeng Guo, Pingzhuo Liang, Congai Zhen, Jinghui Wang, Lei Zhang, Pei Liang, Xiwu Gao

**Affiliations:** 1 Department of Entomology, China Agricultural University, Beijng 100193, China; 2 College of Agriculture & Food Engineering, Baise University, Baise 533000, China

**Keywords:** *Aphis gossypii*, broflanilide, susceptibility baseline, resistance monitoring

## Abstract

**Simple Summary:**

The control of *Aphis. gossypii* has relied on the application of insecticides, but the resistance to insecticides has become a key factor in the successful management of *A. gossypii*. It is a critical measure to introduce a novel insecticide with a different action mode in the resistance management. We found that broflanilide has high insecticidal activity against *A. gossypii*. Broflanilide susceptibility was determined in field populations of *A. gossypii* from main cotton planting areas of China. Meanwhile, the susceptible baseline of the cotton aphid to broflanilide was established. These results suggested that the cotton aphid possessed high susceptibility to broflanilide. The susceptible baseline provides a comparative basis for the future resistance monitoring in the management of cotton aphids.

**Abstract:**

The *Aphis gossypii* is an important pest that can damage cotton plants and can cause a huge economic loss worldwide. Chemical control is a main method to manage this pest, but the cotton aphid resistance to insecticides has become a severe problem in the management of the cotton aphid. It is important to introduce a novel insecticide for rotational application with other insecticides. Broflanilide, as a meta-diamide insecticide with a special mode of action, showed high efficiency against lepidopterous larvae. However, we found that broflanilide possessed high insecticidal activity against the sap-sucking pest *A. gossypii*. The susceptibility of *A. gossypii* to broflanilide from 20 field populations in main cotton planting areas of China in 2021 was determined by the leaf-dipping method. LC_50_ values of broflanilide to *A. gossypii* ranged from 0.20 μg mL^−1^ to 1.48 μg mL^−1^. The susceptible baseline of *A. gossypii* to broflanilide was established with the LC_50_ value of 0.41 μg mL^−1^ and might be used to calculate the resistance ratio (RR) of cotton aphid population in broflanilide resistance monitoring. The RR value of field populations in China was from 0.49 to 3.61 in 2021. It suggested that the broflanilide may be a potential agent in the resistance management of *A. gossypii* to insecticides. These results are significantly useful for the rational chemical control of cotton aphids.

## 1. Introduction

Broflanilide is a novel insecticide with a typical meta-diamide [3-benzamido-N-(4-(perfluoro propane-2-yl) phenyl) benzamide] structure. It is categorized as a member of a new group (Group 30) by the Insecticide Resistance Action Committee (IRAC) [1]. Broflanilide is metabolized to the active desmethyl-broflanilid in insect pests, which is a noncompetitive antagonist to act on the third transmembrane domain of γ-aminobutyric acid receptor (GABAR) [2,3]. Broflanilide was effective against larvae of Lepidoptera and Coleoptera, thrips and sanitary pests, as well as *Myzus persicae* (Hemiptera: Aphididae) and *Aphis fabae* (Hemiptera: Aphididae) [4,5,6,7,8]. Broflanilide has exhibited low toxicity to the major natural enemy *Cyrtorhinus lividipennis* (Hemiptera: Miridae) in paddy fields [9]. We found that broflanilide possessed high activity against *A. gossypii*, which has developed very high resistance to neonicotinoid and pyrethroid insecticides in China [10,11,12,13].

The cotton aphid, *A. gossypii* Glover (Hemiptera: Aphididae), is a sap-sucking pest that threatens a wide range of crops, including pepper, tomato, eggplant, watermelon, cucumber, squash, pumpkin, citrus fruit, potato, and cotton [14]. It can damage the hosts by direct sap-feeding plant tissue nutrition and indirectly through the transmission of plant pathogens [15]. The control of cotton aphids is largely dependent on the use of insecticides. The cotton aphid has evolved high level of resistance to many insecticides, including organophosphates [10,16], carbamates [17], pyrethroids [11], and neonicotinoids [18,19], because of their continuous use for a long time.

Organophosphates and carbamates were widely used to control cotton aphids in 1960s–1980s in China, but the cotton aphid evolved 23 and 148 fold resistance to two organophosphate insecticides, parathion and demeton, in North China in 1964 [20]. The resistance of cotton aphids to omethoate increased to 60–80 times in four regions of Shandong province in 2004 compared with 22–37 times in 1985 [17]. The cotton aphid resistance to omethoate in six areas of Xinjiang reached 2137–9501 fold in 2018 [21]. In 1999, the cotton aphid evolved 18–34 times resistance to a carbamate insecticide, carbosulfan, in Shandong province [17]; however, the resistance of the cotton aphid from Xinjiang reached 148.0 times in 2018 [21]. Pyrethroid insecticides were used for the control of cotton aphids since the mid-1980s, but the resistance of the cotton aphid to pyrethroids was rapidly detected [20]. In 1985, the cotton aphid developed 3228 and 241 fold resistance to deltamethrin and fenvalerate [20]; by 2013, the field populations of the cotton aphid evolved more than 1000 times resistance to beta-cypermethrin in China [11]. In 2018, the cotton aphid developed 353–4932 times resistance to beta-cypermethrin in cotton fields of Xinjiang [21]. At present, neonicotinoid insecticides are widely applied for the control of cotton aphids; however, *A. gossypii* also evolved high resistance to neonicotinoids including imidacloprid, acetamiprid, and thiamethoxam, and the resistance ratio reached more than 471.2-fold in Shandong and Shanxi provinces and the field population in Xinjiang developed 1095 times resistance [21,22].

The insecticide resistance has become a critical problem for the successful management of cotton aphids. The application of the insecticides with new structural types is one of the important measures for the control of *A. gossypii* and resistance management in fields. The broflanilide displayed a high efficiency against 20 field populations of the cotton aphid in 2021. We established the susceptible baseline of *A. gossypii* to broflanilide as a reference for future resistance monitoring.

## 2. Materials and Methods

### 2.1. Insects

**Figure 1:**
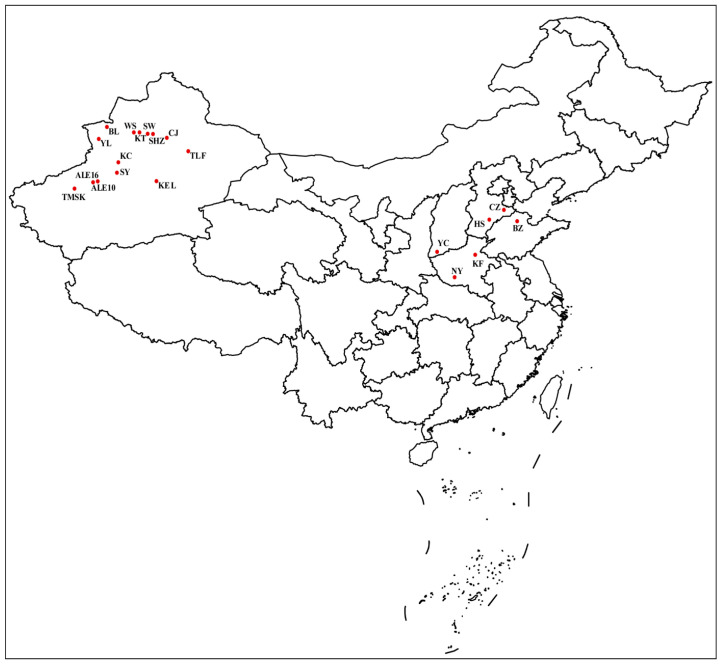
Geographical distribution of *Aphis gossypii* samples in China.

The field populations of *A. gossypii* were collected from 20 main cotton planting areas of China from July to September in 2021. More than 2000 apterous aphids were randomly collected according to a five-point sampling method from 20–30 cotton plants at each sample site to ensure that the samples were representative. The information and geographic distribution of the field populations collecting information are shown in Figure 1 and Table 1. Neonicotinoids (imidacloprid, acetamiprid, and thiamethoxam), pyrethroids (beta-cypermethrin and deltamethrin), and a sulfoximine insecticide (sulfoxaflor) have been used in these regions for the control of cotton aphid. These cotton aphid populations were transferred to the laboratory and reared on cotton seedlings (*Gossypium hirsutum* L. var. Xinmian No. 1) without pesticide exposure. All field populations were reared in insectaria under the controlled conditions with 22 ± 1 °C, 60–70% of relative humidity, and 16:8 h (L:D) of photoperiod. The aphid populations were raised for at least 3 generations in the insectaria and used for subsequent experiments.

### 2.2. Chemicals

Broflanilide was obtained from Badische Anilin-und-Soda-Fabrik (BASF, Beijing, China) with 98% purity. Analytical grade acetone (>99.5% purity) and agar strips (>99% purity) were purchased from Sinopharm Chemical Reagent Co., Ltd. (Beijing, China), and Triton X-100 was obtained from Sigma-Aldrich Co. (Saint Louis, MO, USA).

### 2.3. Toxicity Bioassays

The toxicity of broflanilide to the cotton aphid was determined by the leaf-dipping method as previously described [23] with slight modifications. We used a two-step dilution method. First, the stock solution of broflanilide (5000 μg mL^−1^) was prepared with acetone for easy dilution with water. The desired concentrations (0, 0.1, 0.1, 0.2, 0.4, 0.8, 1.6, 6.4, 10.0 μg mL^−1^) were obtained by diluting the above stock solution with the distilled water with 0.05% triton-X 100 before bioassay. Fresh cotton leaves were cut into 21 mm-diameter leaf discs with punch, and then these leaf discs were immersed in the above diluted solutions for 15 s. The leaf discs only treated with 0.05% (*v*/*v*) Triton-X 100 water were used as the corresponding control. The treated leaf discs were placed indoors to dry, and then the dried leaf discs were put into 12-well cell plates that contained 2.5 mL of 1.85% (*w*/*v*) agar. Healthy apterous adult aphids were gently transferred into 12-well cell plates from cotton seedlings using a soft small brush, and then the plate was sealed with Chinese art paper to prevent aphids from escaping, three replicates per concentration and at least 25 aphids in each well. The 12-well cell plates were placed under the same condition as the aphid culture. The number of live and dead aphids was scored after 72 h exposure. The aphid was considered dead if it could not move by the touch of a soft small brush.

### 2.4. Data Analysis

Probit analysis was used to calculate the slope of the regress curve, LC_50,_ and LC_90_, and 95% confidence limits by POLO Plus 2.0 software [24], and the Chi-square (*χ*^2^) values and degrees of freedom (*df*) were obtained from this software. The *p*-value was calculated by the CHIDIST function of Excel 2019 using Chi-square values and degrees of freedom.

The bioassay data of all aphid populations were pooled for the establishment of the susceptible baseline of *A. gossypii* to broflanilide, and the susceptible baseline was used to calculate the resistance ratio by LC_50_ of field population/susceptible baseline.

## 3. Results

### 3.1. The Toxicity of Broflanilide to Field Populations of Aphis gossypii

Broflanilide exhibited high toxicity against all field populations of *A. gossypii* with LC_50_ values of 0.20–1.48 μg mL^−1^ and LC_90_ values of 0.70–4.90 μg mL^−1^ (Table 2). The slope ranged from 1.24 ± 0.12 to 6.59 ± 1.10 for field populations of *A. gossypii* (Table 2). It suggested that there was higher susceptible consistency among individuals of cotton aphid population. The ALE10 population was the most susceptible to broflanilide with the LC_50_ value of 0.20 μg mL^−1^, and the population with the largest LC_50_ value was from KC, LC_50_ value of 1.48 μg mL^−1^. The difference of LC_50_ values between ALE10 and KC populations was 7.4 times.

### 3.2. Susceptible Baseline of Aphis gossypii to Broflanilide

**Figure 2:**
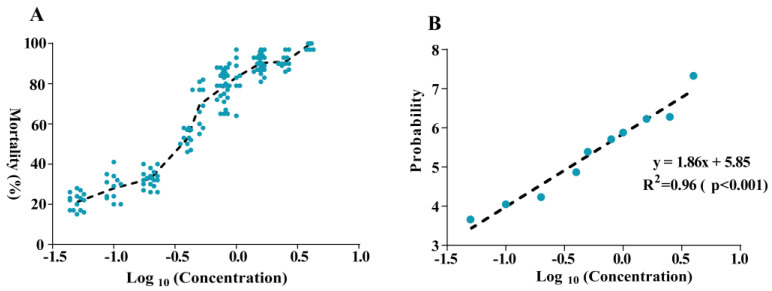
The dose-mortality curve and toxicity regression of broflanilide against cotton aphids. (**A**): The dose-mortality curve of broflanilide to *Aphis gossypii*. The concentration logarithm as X-axis and mortality as Y-axis. (**B**): The toxicity regression curve of broflanilide to *Aphis gossypii*. The concentration logarithm as X-axis and mortality probability value as Y-axis. The correlation coefficient is 0.96 (*p* < 0.001).

The curve of dose-mortality that was used to calculate the susceptible baseline of *A. gossypii* to broflanilide showed an S-shaped distribution (Figure 2A). The toxicity regression analysis showed the R^2^ = 0.96 (*p* < 0.001) and slope =1.86 (Figure 2B), which indicated a high linear relationship between concentration logarithm and mortality probability value. 

We established that the susceptible baseline of *A. gossypii* to broflanilide for the LC_50_ value of 0.41 μg mL^−1^, and the LC_90_ was 1.63 μg mL^−1^ and slope was 2.12 ± 0.08 (Table 2). The susceptible baseline was used to calculate the resistance ratio (RR) of cotton aphid populations to broflanilide. All field populations in 2021 were susceptible to broflanilide, and the RR ranged from 0.49 to 3.61.

## 4. Discussion

The application of chemical insecticides is an indispensable measure in the practice of cotton pest management. The resistance of cotton aphids to traditional insecticides (organophosphorus, carbamate, pyrethroid, and neonicotinoid insecticides) has become a constraint in the control of cotton aphids [10,13,19]. It is an essential way for pest management to rotate application among different action modes of insecticides. Broflanilide has a novel mode of action, classified as a new member of group 30 (mode of action: GABA-gated chloride channel allosteric modulator) [1,25]. As an antagonist of GABAR, broflanilide exhibited high biological activity against various insect species such as *Helicoverpa armigera* (Lepidoptera: Noctuidae), *Spodoptera exigua* (Lepidoptera: Noctuidae) [26], *Spodoptera Litura* (Lepidoptera: Noctuidae) [27], and *Anopheles arabiensis* (Diptera: Culicidae) [28], which also seriously affected the growth and development of some pests [29,30]. Interestingly, broflanilide possessed low toxicity to some natural enemies such as *C. lividipennis* and *Typhlodromips swirskii* (Acari: Phytoseiidae) and have low residue in the environment [9,31,32].

In this study, broflanilide showed high biological activity to *A. gossypii* with the LC_50_ values ranging from 0.20 to 1.48 μg mL^−1^ for all field populations in cotton production areas of China. Broflanilide has similar toxicity to *A. fabae* and *Mythimna separata* (Lepidoptera: Noctuidae) with the LC_50_ of 0.15 μg mL^−1^ and 0.64 μg mL^−1^, respectively [7]. Xu et al. (2020) determined that broflanilide at 10 μg mL^−1^ could result in 100% mortality of the *M. persicae*, and the LC_50_ values of broflanilide against the *Plutella xylostella* (Lepidoptera: Plutellidae), *S*. *exigua*, and *Chilo suppressalis* (Lepidoptera: Crambidae) were only 0.13, 0.92, and 1.23 μg mL^−1^, respectively [8]. Tang et al. (2021) determined the susceptibility of *H. armigera* and *S. exigua* collected from Hunan province to broflanilide, and the LC_50_ values were 0.038–0.068 μg mL^−1^ and 0.039–0.087 μg mL^−1^ [26]. The lethal activity of broflanilide against third-instar larvae and adults of *S. litura* were 0.13 mg kg^−1^ (LD_50_) and 3.59 μg mL^−1^ (LC_50_), respectively [27].

The susceptible baseline of broflanilide against *A. gossypii* was established by pooling the bioassay data of all field populations with the LC_50_ of 0.41 μg mL^−1^. According to research reports, LC_50_ values of the susceptible baseline for *A. gossypii* were 0.50 μg mL^−1^ for beta-cypermethrin and 1.1 μg mL^−1^ for deltamethrin [11]. LC_50_ values of sulfoxaflor and imidacloprid against the susceptible strain were 0.64 μg mL^−1^ and 0.32 μg mL^−1^, respectively [31,33]. Shi et al. (2022) also used the mean LC_50_ value (0.149 μg mL^−1^) of 16 field populations as the susceptible baseline of *A. gossypii* to afidopyropen [34]. Our results demonstrated that *A. gossypii* has a high susceptibility to broflanilide, which is similar to the susceptible baseline of the insecticides mentioned above.

*A. gossypii* has developed high resistance to some commonly used insecticides, such as pyrethroids and neonicotinoids [10,11,12]. Detoxifying enzymes including esterases and cytochrome P450 monooxygenases (P450) and point mutations in sodium channels have been demonstrated to contribute *A. gossypii* resistance to pyrethroids [11,35]. Both the enhancement of P450 activity and target mutations in the nicotinic acetylcholine receptors were involved in *A. gossypii* resistance to neonicotinoids [19,36]. Broflanilide, as a noncompetitive antagonist of targeting on GABAR, displayed high efficiency to field populations of *A. gossypii*, although *A. gossypii* has developed high resistance to pyrethroids and neonicotinoids in cotton fields. Similarly, *A. arabiensis* was susceptible to brofilanilide, but it possessed high resistance to pyrethroids [28]. In addition, the *P. xylostella* has developed 1143-fold resistance to abamectin [37], and *H*. *armigera* evolved 20.36 and 39.12 fold resistance to chlorantraniliprole and benzoate, but broflanilide still showed high insecticidal activities against their larvae [26]. This indicated that broflanilide did not exhibit the cross-resistance with other insecticides mentioned above.

## 5. Conclusions

Broflanilide exhibited good biological activity against *A. gossypii*, which provides a potential alternative for the control of cotton aphids. These results are useful for the chemical control of *A. gossypii* in cotton fields.

## Figures and Tables

**Table 1:** Information of *Aphis gossypii* field populations used for bioassay in China.

Populations	Location (City, Province)	Longitude and Latitude	Collecting Date
CZ	Cangzhou, Hebei	116.87° E, 38.31° N	3 September 2021
HS	Hengshui, Hebei	115.58° E, 37.55° N	21 July 2021
KF	Kaifeng, Henan	114.35° E, 34.79° N	10 September 2021
NY	Nanyang, Henan	112.54° E, 33.00° N	2 September 2021
BZ	Binzhou, Shandong	118.02° E, 37.43° N	6 August 2021
YC	Yuncheng, Shanxi	111.00° E, 35.02° N	5 August 2021
KEL	Kuerle, Xinjiang	86.39° E, 40.59° N	30 July 2021
ALE10	Alaer10, Xinjiang	81.24° E, 40.56° N	30 July 2021
ALE16	Alaer16, Xinjiang	80.84° E, 40.50° N	30 July 2021
BL	Bole, Xinjiang	82.05° E, 44.85° N	13 July 2021
CA	Changji, Xinjiang	87.31° E, 44.01° N	13 July 2021
KC	Kuche, Xinjiang	83.05° E, 42.08° N	30 July 2021
KT	Kuitun, Xinjiang	84.90° E, 44.43° N	13 July 2021
SW	Shawan, Xinjiang	85.62° E, 44.33° N	13 July 2021
SY	Shaya, Xinjiang	82.92° E, 41.25° N	30 July 2021
SHZ	Shihezi, Xinjiang	86.08° E, 44.31° N	13 July 2021
TMSK	Tumushuke, Xinjiang	79.21° E, 40.00° N	30 July 2021
TLF	Tulufan, Xinjiang	89.19° E, 42.94° N	17 September 2021
WS	Wusu, Xinjiang	84.68° E, 44.44° N	13 July 2021
YL	Yili, Xinjiang	81.32° E, 43.92° N	17 September 2021

**Table 2:** Toxicity of broflanilide to *Aphis gossypii* field populations.

Populations	Slope ± SE ^a^	LC_50_ (95%CL) ^b^ μg mL^−1^	RR ^c^	LC_90_ (95%CL) ^b^ μg mL^−1^	*χ^2^*(*df*) ^d^	*p* Value
ALE10	1.58 ± 0.20	0.20 (0.09–0.32)	0.49	1.28 (0.87–2.13)	16.03 (12)	0.19
ALE16	1.24 ± 0.12	0.44 (0.28–0.66)	1.07	4.77 (2.95–9.21)	14.04 (12)	0.30
BL	1.91 ± 0.20	0.45 (0.30–0.62)	1.10	2.13 (1.57–3.16)	17.74 (14)	0.22
BZ	3.02 ± 0.33	0.76 (0.60–0.92)	1.85	2.02 (1.64–2.69)	18.41 (16)	0.30
CJ	2.90 ± 0.37	0.51 (0.38–0.63)	1.24	1.41 (1.15–1.84)	12.01 (16)	0.74
CZ	2.42 ± 0.25	0.37 (0.27–0.46)	0.90	1.25 (0.97–1.75)	14.25 (12)	0.29
HS	4.46 ± 0.69	0.36 (0.26–0.45)	0.88	0.70 (0.56–0.90)	15.72 (16)	0.47
KC	2.47 ± 0.42	1.48 (0.97–1.96)	3.61	4.90 (3.60–8.30)	14.35 (14)	0.42
KEL	4.37 ± 0.75	0.55 (0.45–0.64)	1.34	1.09 (0.91–1.46)	11.56 (12)	0.48
KF	1.71 ± 0.23	0.25 (0.12–0.40)	0.61	1.40 (0.88–3.00)	15.31 (10)	0.12
KT	3.92 ± 0.47	1.10 (0.89–1.30)	2.68	2.34 (1.94–3.04)	15.21 (14)	0.36
NY	2.74 ± 0.46	0.46 (0.27–0.63)	1.12	1.35 (1.00–2.10)	16.03 (14)	0.31
SHZ	2.19 ± 0.49	0.68 (0.14–1.12)	1.66	2.61 (1.78–5.05)	18.47 (13)	0.14
SW	6.56 ± 1.10	0.89 (0.74–1.01)	2.17	1.39 (1.20–1.80)	14.93 (13)	0.31
SY	1.97 ± 0.38	0.42 (0.14–0.65)	1.02	1.90 (1.31–4.12)	18.96 (13)	0.12
TLF	2.02 ± 0.26	0.34 (0.23–0.44)	0.83	1.45 (1.11–2.08)	10.64 (13)	0.64
TMSK	3.21 ± 0.70	0.67 (0.31–0.89)	1.63	1.67 (1.30–2.81)	15.62 (12)	0.21
WS	2.08 ± 0.34	0.60 (0.32–0.86)	1.46	2.47 (1.88–3.42)	10.15 (13)	0.68
YC	2.37 ± 0.33	0.41 (0.24–0.57)	1.00	1.42 (1.05–2.14)	17.94 (15)	0.27
YL	3.69 ± 0.57	0.43 (0.31–0.53)	1.05	0.95 (0.78–1.22)	7.96 (13)	0.85
SBL ^e^	2.12 ± 0.08	0.41 (0.37–0.44)	1.00	1.63 (1.52–1.78)	168.12 (174)	0.61

^a^ SE, Standard Error. ^b^ CL 95%, Confidence Limits of 95%. ^c^ RR, Resistance Ratio = LC_50_ of filed populations/susceptible baseline, the LC_50_ value of the broflanilide susceptible baseline was 0.41 μg mL^−1^; ^d^ *χ*^2^ (*df*), Chi-square (*χ*^2^) and degrees of freedom (*df*); ^e^ SBL, Susceptible Baseline of *Aphis gossypii* to broflanilide.

## Data Availability

The data presented in this study are available in this article.
